# Neutrophil-to-Lymphocyte Ratio as a Prognostic Marker of Functional Outcome in Patients With Intracerebral Hemorrhage (ICH) and Its Comparison With ICH Score: A Hospital-Based Study

**DOI:** 10.7759/cureus.69350

**Published:** 2024-09-13

**Authors:** Suvarthi Ray, Vijay Kumar, Ratnadeep Biswas, Vishnu S Ojha, Divendu Bhushan, Ravi Kirti, Sanjeev Kumar

**Affiliations:** 1 Department of General Medicine, All India Institute of Medical Sciences, Patna, Patna, IND; 2 Department of Emergency Medicine, All India Institute of Medical Sciences, Patna, Patna, IND

**Keywords:** functional outcome, intracerebral hemorrhage, intracerebral hemorrhage score, modified rankin scale, neutrophil-to-lymphocyte ratio, prognosis, stroke

## Abstract

Background: The neutrophil-to-lymphocyte ratio (NLR) signifies systemic inflammation, which may correlate with worse outcomes in intracerebral hemorrhage (ICH) patients. This study explored NLR as a prognostic marker of functional outcomes in ICH and compared it with the ICH score.

Methods: This cross-sectional study was conducted at a tertiary-care hospital in India. Blood was collected from patients with ICH to calculate NLR. Functional outcomes were evaluated using the modified Rankin Scale (mRS) at discharge and 90 days follow-up.

Results: The area under receiver operating characteristic curve (AUROC) for NLR predicting poor mRS scores (3-6) at discharge was 0.695 (p = 0.109), and at follow-up, it was 0.729 (p < 0.001) with a cut-off of ≥7.2, sensitivity 68%, and specificity 72%. The AUROC for ICH score was 0.846 (p = 0.003) at discharge and 0.845 (p < 0.001) at follow-up. DeLong's test indicated the ICH score had significantly better predictive performance than NLR at follow-up (p = 0.018).

Conclusions: NLR is a potential prognostic marker for ICH outcomes, showing significant predictive value at 90 days follow-up. However, the ICH score remains a more reliable predictor. Integrating NLR into the ICH score may enhance its prognostic accuracy, but further validation in multicentric studies is needed.

## Introduction

Stroke is one of the leading causes of disability globally and stands as the second most common cause of death worldwide. According to the Global Stroke Factsheet, the lifetime risk of experiencing a stroke has increased by 50% over the past 17 years, and current estimates indicate that one in four people will suffer a stroke during their lifetime [1]. Despite advances in management, the burden of stroke is increasing every year with an increase in morbidity and mortality, disproportionately affecting the lower and lower-middle-income countries [2,3].

Intracerebral hemorrhage (ICH) constitutes 10-15% of all initial strokes, with an incidence of approximately 29.9 per 100,000 person-years, and is associated with higher mortality and more severe disabilities than other stroke types [4,5]. Hemorrhagic stroke occurs when a blood vessel in the brain ruptures, causing bleeding within the brain tissue. ICH occurs most commonly due to long-standing hypertension, but other causes, such as head trauma and pre-existing lesions, such as vascular malformations or tumors, are often encountered as well [6].

Despite recent improvements in medical intensive care, the mortality and morbidity rates for ICH in intensive care units remain high. ICH carries a significant risk of death, with up to 54% of patients dying within a year [7]. Even among survivors, only a small proportion (12-39%) regain full independence [8]. Thus, the search for more efficient screening modalities and better optimization of management for ICH cases is always going on.

The neutrophil-to-lymphocyte ratio (NLR) is a simple calculation derived from routine blood tests that reflects the balance between inflammation and immune regulation. Higher NLR values have been linked to worse outcomes in various diseases, including heart disease, cancer, and infections [9]. Recent studies have highlighted its prognostic value in predicting outcomes in patients with various conditions, such as COVID-19, rheumatoid arthritis, and cancers like gastric cancer, underscoring its utility in both acute and chronic disease management [10-12].

Some studies have recognized NLR as a potential prognostic marker for ICH, reflecting the inflammatory response associated with this condition. Elevated NLR levels have been linked to worse outcomes in ICH patients, including higher mortality and greater disability [13]. However, research on NLR as a prognostic tool for ICH is still in its early stages, with few studies published, particularly in developing countries like India, where stroke incidence is high and healthcare resources are often limited [14]. More extensive studies are needed in these settings to validate the reliability and utility of NLR in predicting the prognosis of ICH patients and to explore its potential role in guiding treatment strategies.

This study aimed to evaluate whether NLR could serve as an independent predictor of patient functional outcomes after intracerebral hemorrhage, in comparison to the standard ICH score.

## Materials and methods

Study setting

This was an analytical cross-sectional study conducted in the inpatient departments of General Medicine, Emergency Medicine, and Trauma at the All India Institute of Medical Sciences (AIIMS), Patna, which is a tertiary-care hospital in Eastern India, for a period of six months.

Study participants

All adult patients presenting to the trauma and emergency departments with acute non-traumatic intracerebral hemorrhage (ICH) were considered for the study. The participants were selected based on the following inclusion and exclusion criteria.

The inclusion criteria for the study required that patients were 18 years of age or older, presented within 24 hours of stroke symptom onset, and had evidence of a hemorrhagic stroke confirmed by a non-contrast computed tomography (NCCT) of the whole brain.

The exclusion criteria included a history of head trauma, fever, or any signs of generalized or localized infection within the previous four weeks, a documented history of previous ICH, current use of anticoagulant medication, presence of subdural or epidural hematoma, known arteriovenous malformation, or patients who had declined consent.

Sampling technique

Total enumeration; all eligible patients within the study period were included into the study.

Study procedure

Patients diagnosed with acute ICH based on computed tomography (CT) scan results were enrolled in the study after meeting specific criteria and providing informed consent. Detailed clinical examinations were conducted to assess various parameters and outcomes. Routine blood investigations, including complete blood counts with white blood cell differentials, were sent.

The information collected included age, gender, comorbidities, NCCT findings such as volume of intracerebral bleed (in milliliters) and presence of intraventricular extension or infratentorial bleed, and Glasgow Coma Scale (GCS) scores. The NLR, a marker of inflammation, was determined by dividing the absolute neutrophil count by the absolute lymphocyte count at the time of initial presentation in the emergency department. The ICH score, a measure of intracranial hemorrhage severity, was assessed using clinical observations and CT scan results.

The modified Rankin Scale (mRS) scores (primary outcome measures of functional disability) were calculated on the day of the discharge and at 90 days post-discharge follow-up via direct follow-up consultation or video conferencing.

Study tools

The modified Rankin Scale is a pre-validated, standard scale ranging from a score of zero, signifying no symptoms at all, to a score of six, signifying death, with higher scores representing worse global disability in stroke patients [15]. mRS scores can be dichotomized into two categories: good (1-2) and poor (3-6), reflecting the functional outcomes.

The NLR is classified into five levels based on the ratios themselves: normal (0-3), grey zone (3-6), mild (6-9), moderate (9-18), and severe (≥18). Higher ratios correspond to increased systemic inflammation and are associated with poorer clinical outcomes [16].

The ICH score is a standardized tool used to assess the severity of intracerebral hemorrhage. It considers factors like patient age, consciousness level (GCS), location and size of the bleed, and whether it extends into the brain's fluid-filled spaces. Higher ICH scores indicate more severe conditions and a higher risk of death [17].

Statistical analysis

Data was collected and analyzed using IBM SPSS version 26.0 (IBM Corp., Armonk, NY, USA). Descriptive statistics were calculated for both categorical and continuous variables.

The ability of NLR and ICH score to predict poor mRS category on discharge and follow-up was assessed using receiver operating characteristic (ROC) analysis. DeLong's test was used to compare the area under the ROC curve (AUROC) of the predictors in case they were significant. A p-value less than 0.05 was considered significant.

Ethical considerations

The study was conducted in accordance with ethical guidelines and approved by the Institutional Ethics Committee of AIIMS Patna (Ref. No. AIIMS/Pat/IEC/PGTh/July21/21). Written informed consent was obtained from each participant or their next of kin if the participant was unable to give consent.

## Results

The study included a total of 120 patients. The majority, i.e., 84 (70%) of them were between the ages of 51 and 80 years. About 74 (61.7%) were males. Hypertension was the most common co-morbidity, affecting 107 (89.2%) patients with ICH, followed by diabetes, which affects 55 (45.8%).

The mean (SD) NLR on admission was 9.34 (6.94), with values ranging from 1.2 to 54. Eleven (9.2%), 39 (32.5%), and 26 (21.7%) patients had a severe, moderate, and mild NLR, respectively. A majority of 36 (30%) patients had an ICH score of two, followed by 31 (25.8%) who had a score of zero, and 29 (24.2%) who had an ICH score of one (Table 1).

**Table 1:** Distribution of neutrophil-to-lymphocyte ratio (NLR) and intracerebral hemorrhage (ICH) scores on admission (N = 120).

Variable	Categories	Count (n)	Percentage (%)
Neutrophil-to-lymphocyte ratio (NLR)	Normal (1-3)	15	12.5%
	Gray zone (3-6)	29	24.2%
	Mild (6-9)	26	21.7%
	Moderate (9-18)	39	32.5%
	Severe (>18)	11	9.2%
Intracerebral hemorrhage (ICH) score	0	31	25.8%
	1	29	24.2%
	2	36	30.0%
	3	17	14.2%
	4	7	5.8%

It was observed that on the day of discharge (discharge), 114 (95.0%) patients had a poor mRS score (3-6). At 90-day follow-up, 80 (66.7%) had a poor mRS score, while 40 (33.3%) patients had a good mRS score (1-2) (Table 2).

**Table 2:** Modified Rankin Scale (mRS) scores at the day of discharge and at 90-day follow-up (N = 120).

Modified Rankin Scale (mRS) category	mRS Score	At discharge		At 90-day follow-up	
		Count (n)	Percentage (%)	Count (n)	Percentage (%)
Good	1	1	0.8%	13	10.8%
	2	5	4.2%	28	23.3%
Poor	3	37	30.8%	25	20.8%
	4	30	25.0%	15	12.5%
	5	24	20.0%	5	4.2%
	6	23	19.2%	34	28.3%

The NLR had a poor ability to predict poor modified Rankin Scale (mRS) outcomes at discharge, with an area under the receiver operating characteristic curve (AUROC) of 0.695 (95% confidence interval: 0.411-0.979) and no statistical significance (p = 0.109). While an NLR cut-off of 4.5 yielded a sensitivity of 81% and specificity of 67% for predicting poor mRS, this cut-off is unreliable due to the overall poor performance of the NLR.

**Figure 1:**
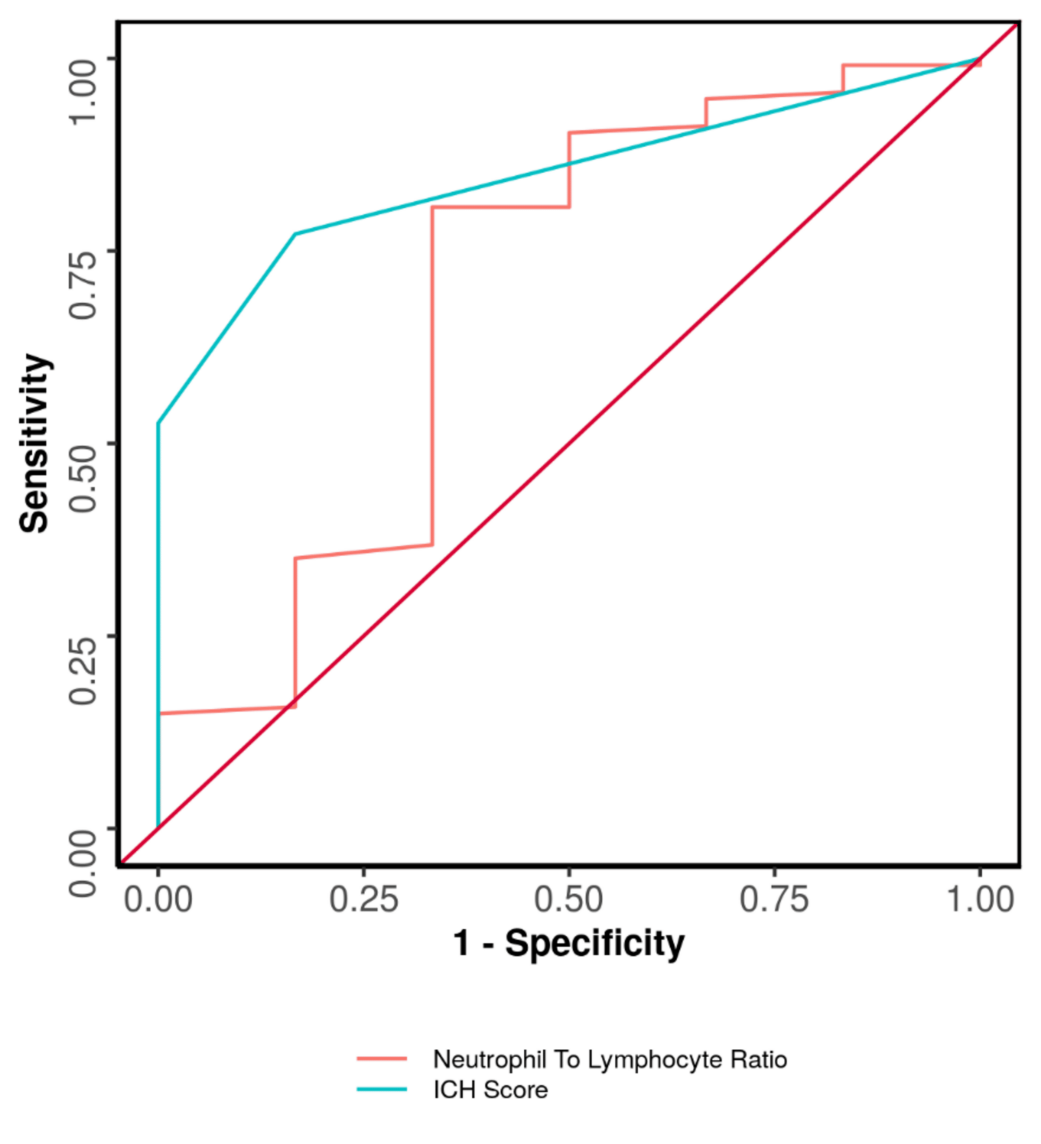
Receiver operating characteristic curve of NLR and ICH score in predicting poor mRS category on the day of discharge. ICH: intracerebral hemorrhage, mRS: modified Rankin Scale, NLR: neutrophil-to-lymphocyte ratio.

In contrast, the ICH score demonstrated good predictive ability for poor mRS at discharge, with an AUROC of 0.846 (95% confidence interval: 0.758-0.935) and statistical significance (p = 0.003). An ICH score cut-off of one achieved a sensitivity of 77% and specificity of 83% for predicting poor mRS (Table 3, Figure 1).

**Table 3:** Comparison of the diagnostic performance of neutrophil-to-lymphocyte ratio (NLR) and intracerebral hemorrhage (ICH) score in predicting poor mRS category on the day of discharge. AUROC: area under receiver operating characteristic curve, PPV: positive predictive value, NPV: negative predictive value, mRS: modified Rankin Scale.

Predictor	AUROC	95% confidence interval of AUROC	p-value	Sensitivity	Specificity	PPV	NPV	Diagnostic accuracy
Neutrophil-to-lymphocyte ratio (NLR)	0.695	0.411-0.979	0.109	81%	67%	98%	15%	80%
Intracerebral Hemorrhage (ICH) Score	0.846	0.758-0.935	0.003	77%	83%	99%	16%	78%

The NLR showed a moderately good ability to predict poor functional outcomes at 90 days, with an area under the curve (AUC) of 0.729. This was statistically significant. Using a cut-off of NLR greater than or equal to 7.2 correctly identified 68% of patients with poor outcomes and 72% of patients without poor outcomes.

**Figure 2:**
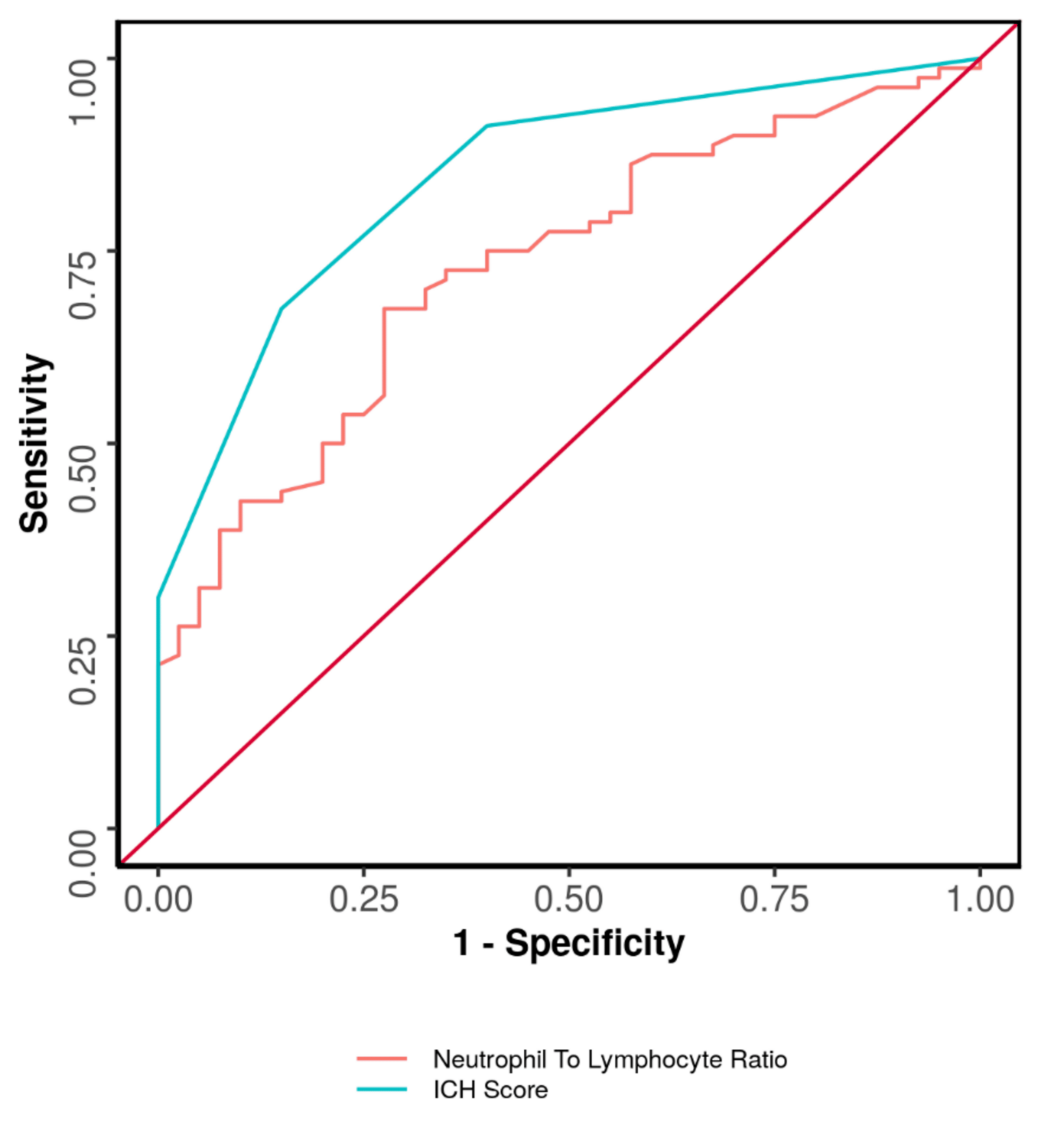
Receiver operating characteristic curve of NLR and ICH score in predicting poor mRS category at 90-day follow-up. ICH: intracerebral hemorrhage, mRS: modified Rankin Scale, NLR: neutrophil-to-lymphocyte ratio.

The ICH score demonstrated a strong ability to predict poor functional outcomes at 90 days, with an AUC of 0.845. This was also statistically significant. An ICH score of two or higher correctly identified 68% of patients with poor outcomes and 85% without poor outcomes (Table 4, Figure 2).

**Table 4:** Comparison of the diagnostic performance of neutrophil-to-lymphocyte ratio (NLR) and intracerebral hemorrhage (ICH) score in predicting poor mRS category at 90-day follow-up. AUROC: area under receiver operating characteristic curve, PPV: positive predictive value, NPV: negative predictive value, mRS: modified Rankin Scale.

Predictor	AUROC	95% confidence interval of AUROC	p-value	Sensitivity	Specificity	PPV	NPV	Diagnostic accuracy
Neutrophil-to-lymphocyte ratio (NLR)	0.729	0.637-0.821	<0.001	68%	72%	83%	53%	69%
Intracerebral hemorrhage (ICH) score	0.845	0.778-0.913	<0.001	68%	85%	90%	57%	73%

The predictors were arranged in descending order of AUROC, and DeLong’s test was performed to compare the AUROC at 90-day follow-up. It was observed that the diagnostic performance of the ICH Score (AUC = 0.845) was significantly better than that of NLR (AUC = 0.729) with a DeLong's Test p-value of 0.018 (Table 5).

**Table 5:** Pairwise comparison of intracerebral hemorrhage (ICH) score and neutrophil-to-lymphocyte ratio (NLR) for predicting poor mRS category at 90-day follow-up. Green: The predictive ability of the row parameter is significantly better than that of the column parameter. mRS: modified Rankin Scale.

Parameter	1	2
1. Intracerebral hemorrhage (ICH) score	-	Green
2. Neutrophil-to-lymphocyte ratio (NLR)		-

## Discussion

This study evaluated the neutrophil-to-lymphocyte ratio (NLR) as a predictor of outcome in patients with intracerebral hemorrhage (ICH) and compared its accuracy to the established ICH score. One hundred twenty patients were included in the analysis. Hypertension was the most common co-morbidity, affecting 107 (89.2%) patients with ICH, followed by diabetes, affecting 55 (45.8%) patients. The AUROC for NLR in predicting poor mRS category on the day of discharge was not statistically significant. It was observed that while both NLR (AUROC = 0.729) and ICH score (AUROC = 0.845) could significantly predict functional outcomes in ICH patients at 90-days follow-up, the ICH score demonstrated superior diagnostic performance (DeLong's Test p-value of 0.018).

This study supports the potential of the NLR as a predictor of long-term outcomes in ICH patients, consistent with previous findings by Lattanzi et al., which reported an association between elevated NLR and poor outcomes at three months post-ICH [13,18,19].

Neutrophils are key players in the acute inflammatory process, responding rapidly to tissue injury by releasing cytokines and reactive oxygen species that can exacerbate brain injury and edema [20]. Conversely, lymphopenia, or a reduction in lymphocytes, often indicates a suppressed adaptive immune response, which is associated with poorer recovery and increased vulnerability to infections [21]. This imbalance between heightened inflammation and weakened immune regulation can lead to secondary brain damage, impaired neuroprotection, and ultimately, worse clinical outcomes in ICH patients [22-24].

It was observed that the prognostic performance of the ICH score was significantly better than NLR. This is in contrary to the findings of Babu et al., who observed that NLR and ICH were both good predictors of functionality with very similar diagnostic performances with AUROCs 0.814 and 0.819, respectively [25]. However, the more robust predictive capability of the ICH score, as demonstrated in this study, suggests it should remain the preferred tool in clinical settings. This finding is consistent with Hemphill et al., who validated the ICH score as a reliable predictor of mortality and functional outcome in ICH patients [26].

The superior performance of the ICH score compared to NLR may be attributed to its comprehensive incorporation of clinical and radiological parameters, which provide a more holistic assessment of intracerebral hemorrhage severity. In contrast, NLR primarily reflects systemic inflammation and does not account for structural or clinical aspects of the hemorrhage, which may limit its predictive accuracy. This difference in scope likely contributes to the observed variance in predictive performance.

Incorporating NLR into the ICH score has the potential to enhance the prognostic accuracy of this widely used tool. The ICH score primarily relies on clinical and radiological parameters only [17]. Adding NLR, an easily obtainable biomarker that reflects systemic inflammation, could provide additional insights into the patient's inflammatory state [27]. By integrating NLR into the ICH score, the new composite score could offer a more comprehensive assessment of both the structural damage and the systemic inflammatory response, potentially leading to better risk stratification and more tailored management strategies for ICH. However, practical considerations such as the standardization of NLR measurement and its consistent application across diverse clinical settings need to be addressed for effective integration.

This study's key strengths lie in its prospective design, comprehensive evaluation of all ICH patients with CT scans, evaluation of functional outcomes at both discharge and after 90 days to assess both the short- and long-term prognostic performance of NLR, and exclusion of patients with infections or those on anticoagulants (which could act as potential confounders by significantly affecting the NLR).

The exclusion of patients with infections or those on anticoagulants may, however, affect the generalizability of our findings, as these exclusions could limit the applicability to patients with varying clinical backgrounds. Additionally, the study was conducted at a single center with a relatively small sample size, which may impact the statistical power and broader applicability of the results. The 90-day follow-up period, while standard, might not fully capture long-term functional outcomes in ICH patients.

Future research should aim to validate these findings in large multicenter studies involving more diverse populations to enhance generalizability. Investigating the combined use of NLR with other inflammatory markers and incorporating NLR into existing scoring stools could provide a more comprehensive and accurate prognostic tool for functional outcomes.

## Conclusions

Although the neutrophil-to-lymphocyte ratio (NLR) demonstrates some prognostic value in intracerebral hemorrhage (ICH), the ICH score remains a more dependable indicator of patient outcomes. Integrating NLR with established scoring systems such as the ICH score to create an integrated new tool could potentially enhance prognostic accuracy, but further validation in larger, multicenter studies is needed. This study adds to the evidence supporting the use of inflammatory markers such as NLR in the prognostic assessment of ICH, and thus, paves the way to improve the management of stroke patients.
